# The Harris Testimonial Fund

**Published:** 1861-01

**Authors:** A. McIlroy, E. C. Rushmore

**Affiliations:** Pres.; Sec.


### Selections.

From the New York Dental Journal.

THE HARRIS TESTIMONIAL FUND.—Pursuant to a call, a meeting of the Dentists of New York and vicinity, took place at the rooms of Messrs. Jones & White, (which were kindly offered for the occasion,) on Monday evening, Oct. 8th, at 8 o'clock.

On motion of Dr. John Allen, Dr. Eleazar Parmly was called to the chair, and Dr. Solyman Brown appointed Secretary; when the Chairman proceeded to make the following remarks in reference to the sad occasion that had called them together:

"*Gentlemen of the Dental Profession*:—I am glad to see so large a number brought together on this occasion, and, permit me, first, to thank you for the honor you have done me, and for your friendly consideration in selecting me to preside at this meeting. There is, perhaps, no one present who has had the same opportunity of knowing the deceased that I have had. My long acquaintance with Dr. Harris in the societies to which we belonged, and my more intimate connection with him in the Baltimore College of Dental Surgery, while I held the office of Provost in that institution, enable me to say, with truth, that I have never known a man of more generous impulses or more genial feelings, than he whose death has awakened our sympathies, and brought so many of the professional brethren together to express their high respect for his professional character, their admiration of his attainments, and their exalted esteem for his moral, social and personal worth.

As one to whom he was long known and endeared by the many virtues that adorned his character, I trust it will not be deemed unbecoming in me to give this testimony concerning one who has labored more arduously as a practitioner, more untiringly as a writer, and more devotedly as a teacher of the Principles and Practice of Dental Surgery, than any person who has in any way, or in any country, ever been connected with our professional art.

In his domestic relations, Dr. Harris was exceedingly happy. His home was one of the most hospitable and delightful that I have ever known. His house and his heart were always open to all those who approached him with the remotest claim upon his benevolence and bounty with a widely extended philanthrophy."

On motion, the Chair appointed a committee of three to draft a series of resolutions expressive of the feeling of this meeting on the present occasion. Drs. Ambler, Brown and Foster were appointed.

While the committee was absent, the Chair suggested that the gentlemen present should all sign the original call for the meeting, and remarked that there were already more names appended to it than there were dentists in the United States when he first commenced practice, in 1815.

The committee returned and reported a Preamble and Resolutions. On motion, the report was accepted by the meeting, and the committee dismissed.

On motion, the meeting proceeded to take up the resolutions *seriatim*, and Nos. 1, 2, 3, and 4 were adopted without dissent. Considerable debate followed the reading of the fifth resolution, and various suggestions and amendments were offered by different gentlemen present. The resolution amended by Dr. Rich, so as to change the number of the committee from twenty to three, was finally adopted by the meeting. The other resolutions were then read and adopted, when, on motion, the report as amended was adopted as a whole, as follows:

PREAMBLE.

Every distinct profession in human society has its leading members, men of energy, talent and eminence. This is as true of the Dental profession as of any other, and not less true in America than in other quarters of the globe.

The names of Greenwood, Wooffendale, Gardette, Hayden, Flagg, Hudson, Koeckor, and Randall, among others that have left their sublunary labors, are evidences of this fact.

It has become our melancholy duty, in pursuance of the objects of this meeting, to add another name to this catalogue more highly distinguished than any of his predecessors, for numerous and valuable contributions to the science and literature of his profession, as well by his writings as by personal inculcations as a teacher at the head of the oldest, and for a long period, the only Dental College in the world.

Chapin A. Harris has gone to his rest. On Sunday, the 30th of the last month he fell asleep to awake in an endless Sabbath. His family, his profession, and his country are left to mourn his loss. We, therefore, offer to this meeting, composed of some of his professional brethren, the following

RESOLUTIONS.

*Resolved, First,* That in the death of Dr. C. A. Harris, removed from his early labors in the prime of manhood; from his family in the fervor of affection; and from his country in the ardor of usefulness; we recognize a sad bereavement, which the members of the Dental profession in all civilized lands have just occasion to deplore.

*Resolved, Second,* That the labors of Dr. Harris, in assisting to establish the American Journal of Dental Science, the American Society of Dental Surgeons, and Baltimore College of Dental Surgery, as well as in the construction and publication of his Dictionary of Medicine and Dentistry, and his Principles and Practice of Dental Surgery, have conferred favors on his profession which gratitude can never repay, because these and other labors in the same direction, have broken his health, abridged his life, and gathered his friends and family around his early grave.

*Resolved, Third,* That a committee of three be appointed by this meeting, to convey to the family of the departed, the sympathy of its members, on occasion of their great and irreparable loss.

*Resolved, Fourth,* That, in view of the great and important services which the self-denying labors of Dr. Harris have rendered to the Dental profession, a suitable testimonial be presented to the family of the deceased, by such members of the profession as may choose to contribute to that object.

*Resolved, Fifth,* That a committee of three be appointed by this meeting to invite all dentists to unite with us in subscribing to raise a money testimonial, to be presented to the widow of the late Dr. Harris, and that this committee have power to appoint sub-committees in Europe and America, to carry out the above object.

*Resolved, Sixth,* That the said committee be instructed to give ample notice of the purpose above explained, not only in all the Dental Journals, but also by means of a printed circular forwarded by mail to all the dentists in the United States, appointing the time for the presentation of said testimonial to the family, and determining the manner in which the fund shall be appropriated to its object.

*Resolved*, *Seventh*, That the committee hereby authorized to receive and appropriate the contributions made for the benefit of the "*Harris Testimonial Fund*," shall be instructed to use no part of said fund for any expenses except for printing and mailing the circulars, and letters sent to members of the Harris family.

*Resolved*, *Eighth*, That this meeting hereby requests all editors of Dental periodicals to publish the proceedings of this meeting, together with the circular issued by the committee, free of charge.

SOLYMAN BROWN,<br>JOHN G. AMBLER,<br>J. H. FOSTER, } *Committee on Resoluions.*

On motion, the committee of three mentioned in the fifth resolution, was appointed, consisting of Drs. Eleazar Parmly, Solyman Brown, and E. J. Dunning. On motion, a committee was appointed to transmit the resolutions adopted by this meeting to the family of the deceased, Dr. Harris. The officers of the meeting, assisted by Dr. Foster, were appointed a committee. A resolution of thanks was then voted to the officers of the meeting, and briefly responded to by the Chair, after which the meeting adjourned.

During the proceedings, Drs. Parmly, Rich, Allen, Hill, Gunning, Roberts, Clarke, Castle, Franklin, Burrows, and others, took occasion to express their sympathies with the occasion which had called them together, and their deep appreciation of the worth of the late Dr. Harris and his labors. The meeting was well attended, some fifty of the most prominent members of the profession in New York being present.

The earnest zeal manifested at this meeting, leaves no room to doubt that a hearty and liberal response will be made to the printed circular of the committee, which will be forwarded in a few days to all the dentists whose address can be obtained, in both hemispheres.

We say in advance to our readers, let the "Harris Testimonial Fund" be such as to fill with joy the heart of the widow and orphans.

At a meeting of the New York Society of Dental Surgeons, held on Wednesday, October 10, the following resolutions were presented by a committee, which had been appointed for that purpose.

*Whereas*, We have learned with the sincerest sorrow, that Professor Chapin A. Harris, of Baltimore, has been stricken down in the midst of his labors, and that the Dental Profession and the civilized world have thus been deprived by death of one of the most devoted and successful pioneers in the cause of science and humanity; and

*Whereas*, We can not allow the death of this great and good man to pass, without endeavoring to give utterance to those feelings which should prevail in the hearts of all men, when one of wisest and most useful of their counselors are taken from their services forever; and

*Whereas*, This sad event has been suitably noticed elsewhere by the whole body of dentists of this city, and will undoubtedly be noticed by the profession throughout the whole country and Europe; therefore,

*Resolved*, That it only remains for us to say, that we will gladly co-operate with our brethren in any way which may be decided upon, to pay a proper tribute of respect and grattitude to the memory of the late highly distinguished Prof. Chapin A. Harris, of Baltimore.

(Signed,) A. McIlroy, *Pres.*
E. C. Rushmore, *Sec.*

